# Impact of the gut microbiota on inflammation, obesity, and metabolic disease

**DOI:** 10.1186/s13073-016-0303-2

**Published:** 2016-04-20

**Authors:** Claire L. Boulangé, Ana Luisa Neves, Julien Chilloux, Jeremy K. Nicholson, Marc-Emmanuel Dumas

**Affiliations:** Metabometrix Ltd, Bio-incubator, Prince Consort Road, South Kensington, London, SW7 2BP UK; Division of Computational and Systems Medicine, Department of Surgery and Cancer, Faculty of Medicine, Imperial College London, Sir Alexander Fleming Building, Exhibition Road, South Kensington, London, SW7 2PH UK

## Abstract

The human gut harbors more than 100 trillion microbial cells, which have an essential role in human metabolic regulation via their symbiotic interactions with the host. Altered gut microbial ecosystems have been associated with increased metabolic and immune disorders in animals and humans. Molecular interactions linking the gut microbiota with host energy metabolism, lipid accumulation, and immunity have also been identified. However, the exact mechanisms that link specific variations in the composition of the gut microbiota with the development of obesity and metabolic diseases in humans remain obscure owing to the complex etiology of these pathologies. In this review, we discuss current knowledge about the mechanistic interactions between the gut microbiota, host energy metabolism, and the host immune system in the context of obesity and metabolic disease, with a focus on the importance of the axis that links gut microbes and host metabolic inflammation. Finally, we discuss therapeutic approaches aimed at reshaping the gut microbial ecosystem to regulate obesity and related pathologies, as well as the challenges that remain in this area.

## The essential role of the gut microbiota in human health

Trillions of microbes live in our guts, which are collectively termed “gut microbiota” [1]. The process of colonization with these microbes starts prenatally, through microbial transmission from mother to fetus [2]. Colonization of the human gut continues after birth and is modulated by factors including gestational age, mode of delivery (natural or by Caesarean section), diet (breastfeeding or infant formula), hygiene, and antibiotic exposure. The environment and diet during the first 3 years of life are crucial to the acquisition of an adult-like microbiota and to the establishment of bacterial–host symbiosis that influences the development of the immune and neurologic systems. The human gut microbiota reaches the characteristics of an adult microbiota between the ages of 2 and 5 years [2].

Gene sequencing data have shown that although a great diversity of bacterial species is found among healthy individuals, the gut metagenome (that is, all the genes in the community of gut microorganisms) is involved in core functions, such as the digestion and degradation of otherwise indigestible nutrients, and the development and stimulation of the immune system and digestive tract of the host [3–7]. The gut microbiota also produces pharmacologically active signaling molecules that interact with the metabolism of the host [8–10]. For example, short-chain fatty acids (SCFAs) are produced by fermentation of dietary fibers by gut bacteria. Their interaction with G protein-coupled receptors (GPCRs) affects insulin sensitivity in adipocytes and peripheral organs, thus regulating energy metabolism [11]. Transient changes in the intestinal ecosystem occur throughout life and in some cases can result in the disruption of microbial–host symbiosis [12]. Owing to the essential role of the gut ecosystem in maintaining host physiology, its alteration can trigger a wide range of physiological disorders, including low-grade inflammation, metabolic disorders, excess lipid accumulation, and loss of insulin sensitivity, which increase the risk of developing metabolic diseases.

**Fig. 1:**
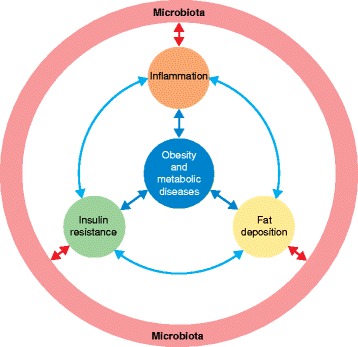
Crosstalk between the gut microbiota and the mammalian host in inflammation and metabolism. The gut microbiota can contribute to host insulin resistance, low grade inflammation, and fat deposition through a range of molecular interactions with the host and therefore can indirectly participate in the onset of obesity and metabolic diseases

Scientific efforts have been focused on understanding the mechanistic basis of the crosstalk between gut microbes and host metabolism in the development and maintenance of host diseases and have revealed the importance of the gut-microbial–host-immune axis [13]. However, whether the presence of keystone bacterial species or the general loss of microbial core functions is the main factor responsible for metabolic and inflammatory disorders of the host is still unclear [13]. In this review, we explore the complex mechanisms that link lipid metabolism, inflammation, insulin signaling, and obesity (Fig. 1). We also discuss the influence of the gut microbiota in the onset of obesity and metabolic diseases through molecular interactions with energy metabolism and inflammation pathways of the host. Finally, we assess the therapeutic potential of manipulating microbial ecology to prevent obesity-related pathologies.

## Obesity and the metabolic syndrome

Obesity is characterized by an excess of adipose tissue and occurs when an imbalance exists between energy intake and energy expenditure [14]. The onset of obesity is a complex process that involves genetic and environmental factors and is often associated with the development of several chronic complications, such as high fasting glucose levels (hyperglycemia), elevated triglyceride levels (hypertriglyceridemia), low levels of high-density lipoprotein (dyslipidemia), and high blood pressure (hypertension) [15]. Individuals who meet at least three of these criteria are clinically diagnosed as having the metabolic syndrome [15], which increases the risk of developing metabolic diseases such as type 2 diabetes and cardiovascular diseases. Most of the individuals with the metabolic syndrome have abnormal fat accumulation, which suggests that the excess of adipose tissue has a causative role in this syndrome [16]. However, this hypothesis has been challenged because several epidemiological studies have identified people with a healthy body mass index (BMI) who nevertheless presented with markers of metabolic dysfunction, such as high levels of triglycerides and accumulation of fat in the liver [15, 17]. The metabolic syndrome should be considered as a clinical diagnosis that is mechanistically driven by a complex combination of factors including impaired fat accumulation, insulin action, and immunity [18].

### Link between impaired insulin action, low-grade inflammation, and obesity

In healthy individuals, insulin triggers glucose uptake in peripheral organs and the secretion of this hormone is activated by the rise in postprandial plasma glucose concentration. Insulin enables the use of extracellular glucose by the body, which results in increased glycolysis and respiration, but it also enables the storage of glucose and lipids by stimulation of glycogenesis and lipogenesis and enables protein synthesis. Insulin also reduces degradation and recirculation of carbohydrates and lipids by inhibiting gluconeogenesis and lipolysis [19, 20]. Impaired insulin action in peripheral organs results in a loss of sensitivity to insulin, which is also called insulin resistance. Loss of insulin sensitivity triggers fasting hyperglycemia and increases hepatic lipid synthesis, dyslipidemia, hypertension, and fat accumulation in adipose tissues. Thus, insulin resistance is an important factor that initiates some of the features characteristic of the metabolic syndrome [20, 21]. In addition, long-term insulin resistance, which leads to a constant raised level of systemic glucose concentration, is the main driver of type 2 diabetes. The metabolic disorders characteristic of the metabolic syndrome (hyperglycemia, hypertriglyceridemia, dyslipidemia, hypertension) are also associated with activation of the immune system [22]. Excessive calorie intake, increased fat accumulation, and lipotoxicity activate the production of effector molecules (cytokines) and cells that are primarily involved in innate immunity [23, 24]. This production promotes a chronic, low-grade inflammatory status, induces the recruitment and activation of many mature immune cells (including mast cells, macrophages, and dendritic cells) in metabolic tissues and particularly in adipose tissues, and also induces recruitment and activation of other cells, such as adipocytes, that modify the tissue milieu and reinforce the inflammatory process [25, 26]. Cai and colleagues have shown that activation of effector molecules of inflammation contributes to desensitizing insulin signaling pathways [24].

At the molecular level, several mechanisms linking the activation of inflammatory pathways and impaired insulin action come into play: activation of IκB kinase complex, extracellular signal-regulated protein kinases 1 and 2 (ERK1/2), and c-Jun N-terminal kinases (JNKs) in inflammatory tissues in individuals with obesity decreases tyrosine phosphorylation of the insulin receptor substrate (IRS) proteins, leading to an attenuation of insulin signaling [27]. However, activation of JNKs and IκB kinase complex does not affect inflammation in the same way and does not attenuate insulin signaling in all tissues [27, 28]. The production of cytokines such as tumor necrosis factor α (TNF-α) or interleukin (IL)-1β in visceral adipose tissues in rodents and humans affects insulin sensitivity by altering the expression of genes encoding IRS-1, the glucose transporter GLUT4, and PPAR-α [29, 30]. Obesity-related inflammation and impaired insulin action are tightly connected; inflammation leads to impaired insulin action, which in turn contributes to the development of metabolic abnormalities. The emergence of chronic inflammation in individuals with obesity has been suggested to promote the clinical progression of the metabolic syndrome and obesity-related pathologies such as type 2 diabetes and non-alcoholic fatty liver disease (also called hepatic steatosis) [22, 31].

## Interactions between gut microbes and host metabolism in the physiopathology of obesity and the metabolic syndrome

Although genetic variants have been associated with susceptibility to developing obesity and type 2 diabetes, the heritability of these variants is fairly modest. The gut microbiota has recently been recognized as a key environmental factor driving metabolic diseases. In fact, the gut microbiota is even seen as a separate endocrine organ, which is involved, through a molecular crosstalk with the host, in the maintenance of host energy homeostasis and in the stimulation of host immunity [32]. Shifts in gut microbial composition caused by external factors can result in a dramatic alteration of the symbiotic relationship between gut bacteria and the host, which promotes the development of metabolic diseases. In particular, the gut microbiota is believed to contribute to metabolic diseases via stimulation of low-grade inflammation [13].

### The gut microbiota affects calorie harvest and energy homeostasis

A body of evidence shows that the gut microbiota helps to harvest energy and increase host fat storage [33, 34]. Germ-free mice have 40 % less total body fat than conventional mice although they ingest 29 % more calories than their conventionally raised littermates [33]. Germ-free mice also gain less weight than conventionally raised mice and they are protected against diet-induced glucose intolerance and the development of insulin resistance [28]. In addition, fecal microbiota transplanted from conventionally raised mice to germ-free mice triggered a 57 % increase in the amount of body fat and a dramatic increase in hepatic triglyceride levels and insulin resistance without modifying the amount of food consumed [11]. The expression of host genes involved in energy homeostasis, lipid metabolism, and mitochondrial metabolism in different parts of the gut, as well as in the liver and adipose tissues, is markedly different in germ-free mice and conventionally raised mice [35].

**Fig. 2:**
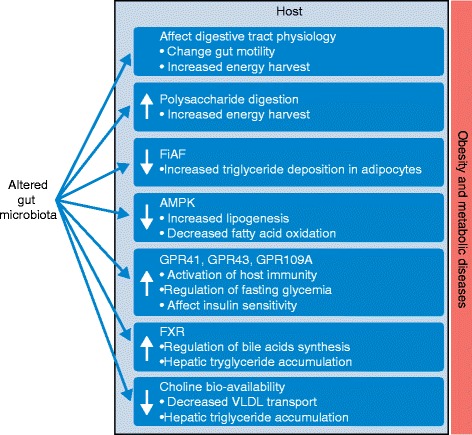
Metabolic and immune interactions between gut microbes and the host in obesity and the metabolic syndrome. The gut microbiota is involved in a molecular crosstalk with the host that modulates host physiology, metabolism, and inflammatory status. In particular, the gut microbiota participates in the physiology and motility of the digestive tract and in the digestion of polysaccharides, which directly influences host energy availability. The gut microbiota inhibits fasting-induced adipose factor (*FIAF*) in the intestine and monophosphate activated protein kinase (*AMPK*) in several organs such as the brain and muscle, which results in increasing fat deposition. The short-chain fatty acids (SCFAs) produced by bacteria from polysaccharides interact with G protein-coupled receptors (GPCRs; GPR41, GPR43, and GPR109A), which stimulates gut motility and host immunity. The gut microbiota also contributes to fat deposition through the regulation of the farnesoid X receptor (*FXR*), the bile acid receptor responsible for the regulation of bile acid synthesis and hepatic triglyceride accumulation. The gut microbiota converts choline to trimethylamine, thus influencing the bioavailability of choline for host use and indirectly affecting phosphatidylcholine production and hepatic triglyceride transport by very-low-density lipoproteins (*VLDL*s)

Studies in germ-free and conventionally raised mice have revealed several mechanisms linking gut bacteria and energy metabolism (Fig. 2):

1. The gut microbiota can have a role in the development of the gut epithelium by increasing the density of small intestinal villi capillaries and by influencing gut physiology and gut motility, thus promoting caloric extraction from the diet [6, 36].
2. Polysaccharides are not digested in the proximal intestine of humans and rodents; instead, they are transformed into digestible compounds such as sugars or SCFAs by the gut microbiota in the distal intestine. These energy substrates are used by colonocytes in particular and the host in general [37].
3. The gut microbiota downregulates the intestinal expression of fasting-induced adipose factor (FIAF), which inhibits lipoprotein lipase in adipose tissues. FIAF activates the breakdown of lipoprotein-contained triacylglycerol into free fatty acids to be used by muscle and adipose tissues. Therefore, the inhibition of FIAF promotes triglyceride deposition in adipocytes [11].
4. The gut microbiota suppresses the release of adenosine monophosphate-activated protein kinase (AMPK), which is primarily expressed in skeletal muscle, brain, and liver in response to metabolic stress (for example, hypoxia, glucose deprivation, exercise). AMPK inhibition promoted by gut bacteria leads to downregulation of mitochondrial fatty acid oxidation, ketogenesis, glucose uptake, and insulin secretion and up-regulation of lipogenesis and cholesterol and tryglyceride synthesis [34, 38].
5. SCFAs are ligands for GPCRs such as GPR41, GPR43, and GPR109A, which are expressed in gut enteroendocrine cells. These specialized cells have essential endocrine functions in the intestine or pancreas. Upon SCFA production, GPCRs stimulate peptide YY (PYY), which leads to changes in gut motility and facilitation of nutrient absorption. Samuel and colleagues [34] showed that GPR41-deficient mice have more lean body mass and less body fat than their wild-type littermates. However, a more recent study had contrasting results, with GPR41 knockout mice showing increased amounts of body fat and decreased energy expenditure in comparison with wild-type mice [39]. GPR43 activation is also thought to reduce fat accumulation and regulate energy metabolism by suppressing insulin sensitivity in adipose tissues and increasing insulin sensitivity in liver and muscle [9, 11]. The gut microbiota also regulates the adaptive immune system in the gut and maintains colonic health in mice through the SCFA-dependent activation of GPR43 [40].
6. Parseus and colleagues [41] proposed that the gut microbiota contributes to the high-fat-diet-induced obesity phenotype through the regulation of the farnesoid X receptor (FXR), the bile acid receptor responsible for the regulation of bile acid synthesis and hepatic triglyceride accumulation.
7. Choline is an essential nutrient for the synthesis of phosphatidylcholine, which is a major component of cell and mitochondrial membranes. Phosphatidylcholine is also a major component of very‐low‐density lipoproteins (VLDL), which are responsible for export of triglycerides to the organs [42]. Defective export of triglycerides by VLDL leads to their accumulation in hepatocytes, which is the central mechanism in the development of hepatic steatosis [43]. The gut microbiota, through its ability to convert choline to trimethylamine, regulates the bioavailability of choline and indirectly affects the storage of triglycerides in the liver [44].

### Shifts in the gut microbial ecosystem in obesity

Human studies and animal models have been used to demonstrate that the gut microbiota is altered in obesity. A comparison of bacterial composition in the gut of lean, wild-type, and obese mice (leptin-deficient *ob*/*ob* mice, in which obesity is induced by a deficiency in leptin, the hormone that controls satiety) showed differences in the abundance of the phyla Bacteroidetes and Firmicutes. In particular, the Firmicutes:Bacteroidetes ratio positively correlated with the obese phenotype independently of diet [45]. Turnbaugh and colleagues [33] also compared the gut microbiota of lean mice and mice with diet-induced obesity and found an increase in the abundance of Firmicutes that was associated with diet-induced obesity. However, the observed differences were related to the growth of a specific class within the Firmicutes phylum, the Mollicutes class, in animals with diet-induced obesity. Moreover, these compositional changes were completely reversed after a return to a normal diet, which suggests that diet is the main contributing factor to obesity-associated changes in the gut microbiota. These observations were supported by the findings of Murphy and colleagues [46], who identified an increase in the Firmicutes:Bacteroidetes ratio in *ob*/*ob* mice and in mice fed a high-fat diet compared with lean mice. Of note, this increase was more significant in the high-fat-diet fed mice than in the *ob*/*ob* mice.

More recently, Ridaura and colleagues [47] have established causal links between gut microbial communities and obesity by transplanting fecal samples from co-twins discordant for obesity into separate groups of germ-free mice. They found that mice colonized with the fecal microbiota of co-twins with obesity had a greater increase in body weight and amount of adipose tissue than the mice colonized with the fecal microbiota of lean co-twins. In addition, obese mice co-housed with lean mice also experienced a lower weight gain than those co-housed with obese mice and a shift in gut microbiota composition towards a lean-like status. In particular, growth of Bacteroidetes was stimulated in obese mice co-housed with lean mice and was associated with the increased expression of proteins involved in branched-chain amino acid catabolism and increased production of SCFAs [47]. It is important to note that although SCFAs are a source of calories for the host, their intestinal production has been mostly associated with reduced inflammation and increased satiety and with overall positive metabolic effects [32, 48]. Altogether, these results show that the lean or obese gut microbial ecosystem in the mouse model is mostly influenced by the diet and to a lesser extent by co-housing with littermates. The effects of co-housing the obese and lean mice were highly transferable in germ-free mice, thus contributing to the protection or the onset of obesity in these mice.

Human studies also indicated an alteration of the gut microbial ecosystem with obesity. Turnbaugh and colleagues [33] observed differences in the distal gut microbiota of individuals with obesity compared to lean individuals and the relative abundance of Bacteroidetes increased as individuals lost weight when undergoing either a fat-restricted or a carbohydrate-restricted low-calorie diet. The decreased Bacteroidetes:Firmicutes ratio found in people with obesity is thought to lead to more efficient hydrolysis of non-digestible polysaccharides in the intestinal lumen and may lead to more calories and fat being extracted from food than occurs in lean individuals [11]. However, other human studies in which gut bacterial composition was compared between lean individuals and individuals with obesity have failed to confirm the association between obesity and a decreased Bacteroidetes:Firmicutes ratio [49, 50]. A recent report has suggested that the microbiota of people with obesity and those who are lean responds differently to the calorie content in the diet [51]. Nutrient absorption induced a shift in the gut microbial composition in lean individuals but not in those with obesity, increasing the relative abundance of Firmicutes while decreasing the relative abundance of Bacteroidetes [50]. Microbial gene richness might also have a role in the inflammatory status of the host, which is related to obesity. Individuals with obesity who have a high bacterial gene count were found to carry a higher proportion of species associated with an anti-inflammatory status (for example, *F. prausnitzii*) and a lower proportion of species associated with a proinflammatory status (for example, *Bacteroides* spp.). Also, the bacterial gene count for genes associated with oxidative stress was higher in individuals with low bacterial gene count than in those with high bacterial gene count [51]. As carrying out a controlled dietary intervention study in humans is difficult, the complex interaction between diet, age, host environment, and host genetic background in the modulation of gut microbial ecosystems is not fully understood. Nevertheless, a recent report suggests that alteration of the gut microbiota by behavioral changes, including new dietary habits [52] and use of antibiotics, could be the main driver of the obesity pandemic [53, 54].

### Chronic inflammation links the gut microbiota to obesity and insulin resistance

**Fig. 3:**
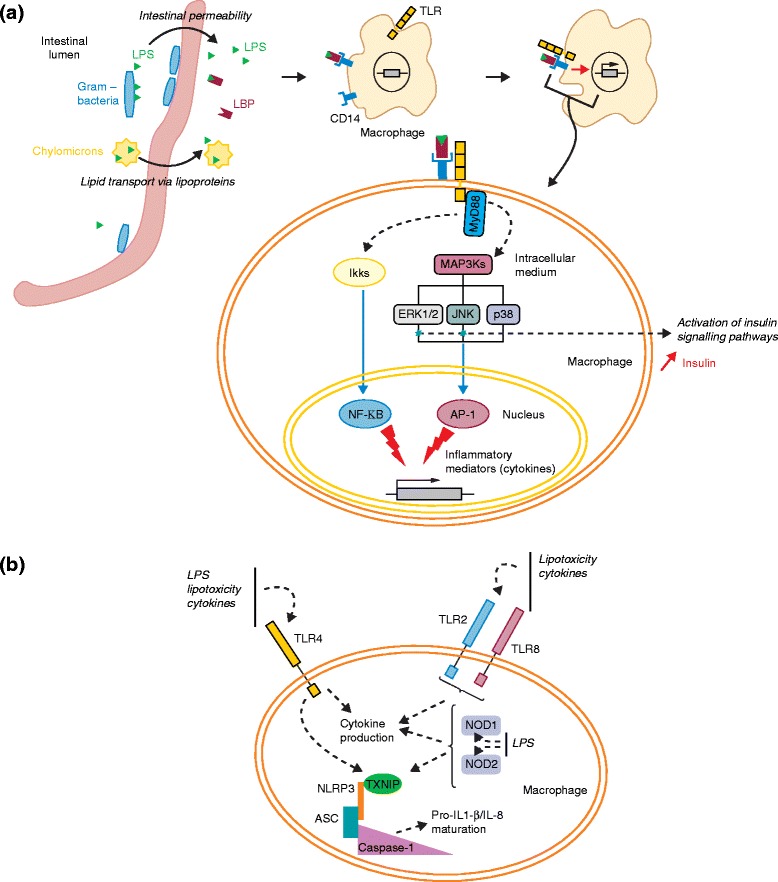
Induction of inflammatory signals in proinflammatory macrophages and their connection with insulin pathways. **a** After translocation of gut bacteria to other tissues, the bacterial lipopolysaccharides (*LPS*) in the circulation and organs activate the transcription of cytokines via Toll-like receptor (*TLR*)4. Activated TLR4 mediates inflammatory signals involving myeloid differentiation primary response gene 88 (*MyD88*)-dependent pathways. The downstream responses trigger the activation of mitogen-activated protein kinase (*MAPK*) pathways, including those involving extracellular signal-regulated protein kinases 1 and 2 (*ERK1/2*), c-Jun-N-terminal kinases (*JNK*), p38, and inhibitor of IκB kinase complex (*IKKβ*). These pathways participate in the activation of transcription factors nuclear factor κB (*NF-κB*) and activator protein 1 (*AP-1*) and cytokine production. ERK1/2 and JNKs are also involved in the induction of insulin signaling pathways. **b** Pattern-recognition receptors such as TLR4, TLR2, and TLR8 are activated by LPS, cytokines, or lipotoxicity. The intracellular nucleotide oligomerization domain (*NOD*)-like receptors also recognize LPS, which leads to induction of thioredoxin-interacting protein (which is encoded by *TXNIP*) and recruitment of other effector molecules such as those that are components of inflammasome pathways [28]. Inflammasomes are multiprotein complexes composed of three proteins: nucleotide-binding domain leucine-rich repeat containing (NLR) protein, adaptor protein ASC, and caspase-1. Inflammasome activation contributes to the maturation of the cytokines interleukin (IL)-1β and IL-8

One of the hallmarks of obesity and obesity-related pathologies is the occurrence of chronic low-grade inflammation [22]. Lipopolysaccharides (LPS), also called endotoxins, which are derived from the outer cell membrane of Gram-negative bacteria, have been thought to initiate the inflammation-related processes associated with the onset of obesity and insulin resistance (Fig. 3) [23]. LPS contain lipid A in their structure and are able to cross the gastrointestinal mucosa via leaky intestinal tight junctions or by infiltrating chylomicrons, the lipoproteins responsible for the absorption of dietary triglycerides and cholesterol from the intestine to the plasma [23, 55, 56]. Once they reach the systemic circulation, LPS infiltrate tissues such as the liver or adipose tissues, triggering an innate immune response [23]. In particular, LPS bind the plasma LPS-binding protein (LBP), which activates the receptor protein CD14 that is located in the plasma membrane of macrophages [56]. The complex thus generated binds Toll-like receptor 4 (TLR4) at the surface of macrophages, which triggers transduction signals that activate the expression of genes encoding several inflammatory effectors, such as nuclear factor κB (NF-κB) and activator protein 1 (AP-1) [56, 57]. LPS also regulate the nucleotide oligomerization domain (NOD)-like receptors present in macrophages and dendritic cells, which cooperate with TLRs to induce NF-κβ. In addition, LPS participate in the recruitment of other effector molecules, such as nucleotide-binding domain leucine-rich repeat containing (NLR) protein, adaptor protein ASC, and caspase-1, which are components of the inflammasome, a multiprotein oligomer that activates the innate immune system [27].

Systemic LPS are found at low concentrations in healthy individuals but reach high concentrations in individuals with obesity, a condition called metabolic endotoxemia [23]. Several mechanisms linking obesity and metabolic endotoxemia have been proposed: during consumption of a high-fat diet, the gut microbiota is modified, which leads to increases in gut permeability and in the systemic levels of bacterial products such as LPS [23]. Additionally, excess fat intake triggers an increase in chylomicrons in the intestine during the postprandial period (following a meal), which favors LPS infiltration into the circulation [58]. Impaired lipoprotein metabolism in patients with type 2 diabetes has also been found to reduce LPS catabolism and might increase endotoxemia-related inflammation [59]. The importance of metabolic endotoxemia in the physiopathology of insulin resistance and obesity has been further highlighted by Shi and colleagues [50], who showed that mice lacking TLR4 were protected against insulin resistance induced by a high-fat diet. Results from another study revealed that LPS infusion into genetically identical male mice for 4 weeks induced a comparable weight gain to that observed in mice consuming a high-fat diet [23]. Furthermore, an interesting animal model, the immunoprotein CD14 knockout *ob*/*ob* mouse, which is unable to induce LPS-mediated inflammatory pathways, was resistant to weight gain and was insulin-hypersensitive, despite being fed with the same diet as leptin-deficient *ob*/*ob* mice [60]. In humans, circulating endotoxin levels were found to increase by 20 % in individuals with obesity or glucose intolerance and by 125 % in individuals with type 2 diabetes compared with the levels in lean individuals [61]. Circulating endotoxin levels were also associated with elevated TNF-α and IL-6 concentrations in adipocytes [62]. In addition, a high-fat or high-carbohydrate diet, but not a diet rich in fiber and fruit, activated systemic LPS secretion, as well as the expression of TLR4, NF-κB, and suppressor of cytokine (SOC) 3, which are factors also involved in pathways that regulate insulin secretion [62]. Together, these results show the important role LPS-mediated inflammatory pathways have in obesity and obesity-related pathologies.

**Fig. 4:**
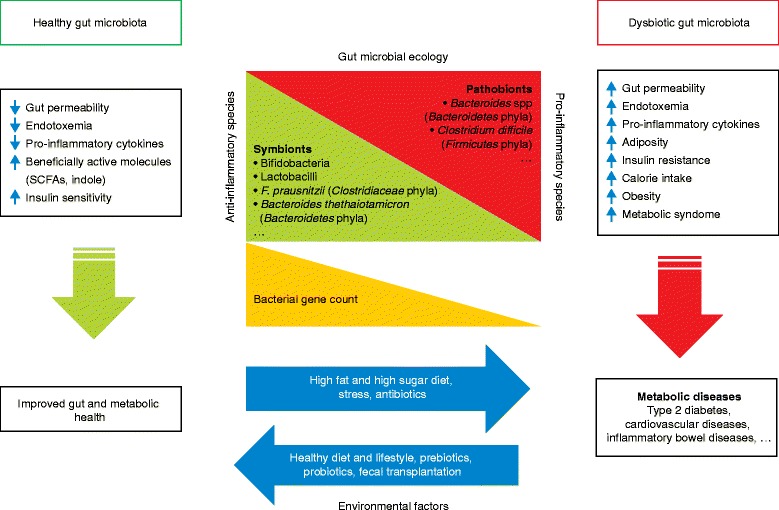
Effects of a healthy gut microbiota and dysbiosis on the gut and metabolic health of the host. A healthy microbiota comprises a balanced representation of symbionts (bacteria with health-promoting functions) and pathobionts (bacteria that potentially induce pathology). A shift toward dysbiosis results from a decrease in symbionts and/or an increase in pathobionts and is likely to be triggered by environmental factors (such as diet, stress, antibiotics, and infections). Low bacterial gene counts have also been associated with altered gut microbial functions and dysbiosis and have been linked to increased fat accumulation, lipopolysaccharide-induced inflammation, insulin resistance, obesity, and the metabolic syndrome. Individuals with these characteristics are more likely to develop metabolic diseases (such as diabetes, cardiovascular diseases, and inflammatory bowel diseases). *LBP* LPS-binding protein, *SCFA* short-chain fatty acid

Other microbial-derived metabolites produced from aromatic amino acids (tyrosine, tryptophan, and phenylalanine) have been suggested to interact with host signaling pathways and thus affect host immunity. Indole was identified as one of the major tryptophan-derived microbial metabolites [63], produced by the action of bacterial tryptophanase (which is present in *Bacteroides thetaiotaomicron*, *Proteus vulgaris*, and *Escherichia coli*, among other species) [64]. Upon absorption, indole can be sulfated in the liver, which results in the production of 3-indoxylsulfate, or can undergo further bacterial metabolism, leading to the production of a range of related compounds, including indole-3-pyruvate, indole-3-lactate, and indole-3-acetate [65]. These metabolites bind human pharmacological targets, which puts the impact of bacterial metabolism of tryptophan in human health and disease into a wider perspective. In particular, 3-indoxylsulfate and indole-3-propionate have been thought to interact with inflammation-related processes in the human host [66]. 3-Indoxylsulfate activates the aryl hydrocarbon receptor (AhR), thus regulating the transcription of IL-6 and several enzymes from the P450 superfamily complex (for example, CYP1A1, CYP1A2, and CYP2S1) [67]. Indole-3-propionate is a pregnane X receptor (PXR) agonist with a beneficial role in gut barrier function, which takes place either through up-regulation of the expression of junctional proteins or by downregulation of TNF-α production in enterocytes [66]. By improving intestinal barrier permeability, indole-3-propionate also indirectly limits the translocation of antigens and pathogens, and LPS infiltration, into the circulation and, therefore, might reduce metabolic endotoxemia and host inflammation [68]. Therefore, a healthy or dysbiotic gut microbiota affects the gut and metabolic health of the host through modulation of gut physiology and LPS infiltration, calorie intake, fat accumulation, and insulin action (Fig. 4).

## Therapeutic potential of manipulating the gut microbial ecology

The study of the metabolic, signaling, and immune interactions between gut microbes and the host, and how these interactions modulate host brain, muscle, liver and gut functions, has raised the concept of therapeutic microbial manipulation to combat or prevent diseases [4, 10]. In particular, the selection of specific gut bacterial strains and the enhancement of the gut microbial ecology represents a promising therapeutic approach to control energy intake and reduce the prevalence of obesity and the metabolic syndrome. Fecal transplantation is an efficient way to reshape the gut microbial ecosystem after antibiotic treatment or to help fight intestinal infection with *Clostridium difficile* and can be used as therapy for inflammatory bowel diseases [69, 70]. A study also showed that nine men with the metabolic syndrome who underwent fecal transplantation with stools from healthy lean individuals had lower fasting levels of triglycerides and developed greater hepatic and peripheral insulin sensitivity after transplantation than nine men who received a transplant of their own stool [71]. Therefore, fecal transplantation may be useful in the struggle against obesity, although the procedure is still at an experimental stage and the mechanisms involved require further understanding.

The use of probiotics and prebiotics to improve the interactions between gut microbes and host metabolism in obesity and other metabolic diseases has been extensively investigated [72]. Probiotics are live microorganisms that, when used as food supplements, beneficially affect the host by improving intestinal microbial balance and changing the composition of the colonic microbiota [73]. Specific bacterial species such as *Bifidobacterium* spp. have been shown to improve glucose homeostasis, reduce weight gain and fat mass, and restore glucose-mediated insulin secretion in mice fed a high-fat diet [73].

Prebiotics are food ingredients that beneficially affect the host by selectively stimulating the growth and/or activity of one or a restricted number of bacteria present in the colon. Prebiotics are composed of oligosaccharides or short-chain polysaccharides. They are found in common dietary products, such as vegetables and whole-grain cereals, and can be added in yoghurt. The best-characterized prebiotics are fructosyl-oligosaccharides (FOS), including inulin (long-chain fructosyl-oligosaccharide), galactosyl-oligosaccharides (GOS), and other oligosaccharides present in milk, which are transformed by the gut microbiota into SCFAs and simultaneously promote proliferation of selected commensal bacteria in the colon [74–77]. For example, inulin has been found to stimulate the growth of bifidobacteria and may reduce caloric intake and fat mass in animals H [75]. Prebiotic stimulation of the growth of bifidobacteria is correlated with increased glucose tolerance, improved glucose-induced insulin secretion, and normalization of inflammation in rodents [78]. GOS also modulate the uptake of monosaccharides from the gut by changing the activity of host monosaccharide transporters, which in turn results in activation of glycolytic pathways [76]. Consumption of prebiotics has also been associated with a reduction in hepatic, renal, and plasma lipid levels in rodents [74, 75]. In particular, GOS supplementation in healthy mice decreased hepatic triglyceride levels by lowering the activity of lipogenic enzymes, fatty acid synthase, and microsomal triglyceride transfer proteins, which are involved in VLDL synthesis [75, 79]. Therefore, ingestion of prebiotics might lower lipogenic activity and increase lipolytic activity.

The effects of prebiotics and probiotics on anti-inflammatory pathways, weight gain, and glucose metabolism in rodents have been largely attributed to SCFA production [37]. SCFAs interact with GPCRs (for example, GPR41 and GPR43) in the immune cells of the human colon and promote expression of specific chemokines in the colonic epithelium [80, 81]. SCFAs repress NF-κB and affect the production of proinflammatory markers, such as IL-2 and IL-10, in leukocytes [82]. SCFAs enhance satiety by increasing the synthesis of PYY and proglucagon in epithelial cells and by inhibiting the expression of neuroendocrine factors such as leptin [83]. Other studies have indicated that the effects of prebiotics on intestinal health and inflammation are also mediated by the secretion of glucagon-like proteins (GLP-1 and GLP-2) in enteroendocrine L cells [77, 84]. Cani and colleagues [68] showed that *ob*/*ob* mice fed a high-carbohydrate diet supplemented with oligofructose have increased intestinal representation of bifidobacteria and lactobacilli, improved connections between tight junctions, lower gut permeability, lower systemic endotoxemia, and lower systemic and hepatic inflammation than *ob*/*ob* mice fed with a high-carbohydrate diet alone. These physiological changes were correlated with GLP-2 levels and disappeared when the mice were treated with a GLP-2 antagonist [68]. Another study also pointed out that a synbiotic treatment combining polydextrose and *Bifidobacterium lactis* B420 lowered the abundance of Porphyromonadaceae in mice fed a high-fat diet [85]*.* This dietary supplement is thought to inhibit T helper 17 (T_h_17) cell infiltration in the small intestine, preventing metabolic inflammation and the development of type 2 diabetes [85].

In humans, probiotic intervention studies have revealed a positive effect of these approaches on glucose metabolism [86]. For example, during a 6-week randomized placebo-controlled study of 60 overweight healthy Indian individuals, the VSL#3 probiotic mix decreased systemic glucose and insulin levels [87]. However, evidence of the anti-obesity effects of prebiotics remain to be demonstrated. Many human studies highlight moderate or no changes in weight loss after prebiotic interventions [88]. Randomized controlled studies have identified surrogate markers of prebiotic treatment (such as plasma PYY, GLP-1, ghrelin) to be negatively correlated with weight gain, inflammation, and impaired glucose metabolism, which support the mechanisms observed in rodents [89, 90]. However, there is no evidence to suggest that prebiotic supplementation in infant formula improves growth or clinical outcomes or causes adverse effects in term infants. Studies in children, adults, and the elderly vary in quality and outcomes. However, prebiotics have been shown to modulate the fecal microbiota and immune function in elderly individuals and to reduce the levels of markers of the metabolic syndrome in overweight adults [91–94]. The effect of prebiotics and probiotics in obesity and related pathologies in humans requires further exploration. In particular, carefully designed studies using appropriate doses of probiotics or prebiotics and controlled diets will be valuable to underpin the individual responses to different types of interventions and their dependence on genetic, environmental, and gut microbial factors.

## Conclusions and future directions

The evidence for a strong contribution of the gut microbiota to the onset of obesity and metabolic diseases is growing. The use of germ-free rodent models has enabled us to establish the molecular basis of the interactions between gut microbes and the physiology of the host. The modifications in the gut microbial ecology by dietary factors, antibiotics, probiotics, or prebiotics that were observed in rodents and humans have further highlighted the key modulatory roles of the gut microbiota and its contribution to host obesity and metabolic diseases. In particular, some metabolic disorders of the host are thought to be associated with an inflammation-related composition of the gut microbiota. However, how external factors (such as diet, stress, age, drug intake, and circadian cycles) affect the gut microbial composition and the effectiveness of microbial functions in rodents and humans is still unclear. In the future, it seems essential to promote top-down analytical approaches on an epidemiological scale, integrating data from dietary questionnaires, data about relevant environmental factors (such as stress or factors that influence circadian rhythms) and history of drug or antibiotic use to understand more deeply the functions of gut bacteria in the physiopathology of human obesity. In combination with animal studies, these integrated epidemiological analyses will enable us to unravel the missing connections within the metabolic axis linking gut microbes and the host and to optimize therapeutic strategies to reshape the gut microbial ecology. Using this knowledge, we also hope to improve the stratification of populations at risk of developing metabolic diseases and offer novel perspectives for personalized healthcare, within which clinicians might be able to tailor therapy on the basis of individual habits and predispositions.

## Abbreviations

AMPK: adenosine monophosphate-activated protein kinase
AP-1: activator protein 1
BMI: body mass index
ERK1/2: extracellular signal-regulated protein kinases 1 and 2
FIAF: fasting-induced adipose factor
FOS: fructosyl-oligosaccharides
FXR: farnesoid X receptor
GOS: galactosyl-oligosaccharides
GPCR: G protein-coupled receptor
IKβ: IκB kinase complex
IL: interleukin
IRS: insulin receptor substrate
JNK: c-Jun N-terminal kinase
LPS: lipopolysaccharide
NF-κB: nuclear factor κB
NLR: nucleotide-binding domain leucine-rich repeat containing
NOD: nucleotide oligomerization domain
PYY: peptide YY
SCFA: short-chain fatty acid
SOC: suppressor of cytokine
TLR: Toll-like receptor
TNF-α: tumor necrosis factor α
VLDL: very-low-density lipoprotein

## References

[1] Bruzzese E, Volpicelli M, Squaglia M, Tartaglione A, Guarino A. Impact of prebiotics on human health. Dig Liver Dis. 2006;38(Suppl 2):S283–7. doi:S1590-8658(07)60011-5. 10.1016/S1590-8658(07)60011-5 17259092

[2] Rodriguez JM, Murphy K, Stanton C, Ross RP, Kober OI, Juge N (2015). The composition of the gut microbiota throughout life, with an emphasis on early life. Microb Ecol Health Dis.

[3] Costello EK, Stagaman K, Dethlefsen L, Bohannan BJ, Relman DA (2012). The application of ecological theory toward an understanding of the human microbiome. Science.

[4] Nicholson JK, Holmes E, Kinross J, Burcelin R, Gibson G, Jia W (2012). Host-gut microbiota metabolic interactions. Science.

[5] Hooper LV, Littman DR, Macpherson AJ (2012). Interactions between the microbiota and the immune system. Science.

[6] Abrams GD, Bishop JE (1967). Effect of the normal microbial flora on gastrointestinal motility. Proc Soc Exp Biol Med.

[7] Rajilic-Stojanovic M, de Vos WM (2014). The first 1000 cultured species of the human gastrointestinal microbiota. FEMS Microbiol Rev.

[8] Nicholson JK, Wilson ID (2003). Opinion: understanding 'global' systems biology: metabonomics and the continuum of metabolism. Nat Rev Drug Discov.

[9] Neves AL, Chilloux J, Sarafian MH, Rahim MB, Boulange CL, Dumas ME (2015). The microbiome and its pharmacological targets: therapeutic avenues in cardiometabolic diseases. Curr Opin Pharmacol.

[10] Nicholson JK, Holmes E, Wilson ID (2005). Gut microorganisms, mammalian metabolism and personalized health care. Nat Rev Microbiol.

[11] Backhed F, Ding H, Wang T, Hooper LV, Koh GY, Nagy A (2004). The gut microbiota as an environmental factor that regulates fat storage. Proc Natl Acad Sci U S A.

[12] Nazli A, Yang PC, Jury J, Howe K, Watson JL, Soderholm JD (2004). Epithelia under metabolic stress perceive commensal bacteria as a threat. Am J Pathol.

[13] Marchesi JR, Adams DH, Fava F, Hermes GD, Hirschfield GM, Hold G (2016). The gut microbiota and host health: a new clinical frontier. Gut.

[14] World Health Organization (WHO). Obesity and overweight. January 2015. http://www.who.int/mediacentre/factsheets/fs311/en/. Accessed 2 April 2016

[15] Alberti KG, Zimmet P, Shaw J (2005). The metabolic syndrome—a new worldwide definition. Lancet.

[16] Despres JP, Lemieux I, Bergeron J, Pibarot P, Mathieu P, Larose E (2008). Abdominal obesity and the metabolic syndrome: contribution to global cardiometabolic risk. Arterioscler Thromb Vasc Biol.

[17] Saito I (2012). Epidemiological evidence of type 2 diabetes mellitus, metabolic syndrome, and cardiovascular disease in Japan. Circ J.

[18] Kahn SE, Hull RL, Utzschneider KM (2006). Mechanisms linking obesity to insulin resistance and type 2 diabetes. Nature.

[19] Perry RJ, Samuel VT, Petersen KF, Shulman GI (2014). The role of hepatic lipids in hepatic insulin resistance and type 2 diabetes. Nature.

[20] Saltiel AR, Kahn CR (2001). Insulin signalling and the regulation of glucose and lipid metabolism. Nature.

[21] Delarue J, Magnan C (2007). Free fatty acids and insulin resistance. Curr Opin Clin Nutr.

[22] Gregor MF, Hotamisligil GS (2011). Inflammatory mechanisms in obesity. Annu Rev Immunol.

[23] Cani PD, Amar J, Iglesias MA, Poggi M, Knauf C, Bastelica D (2007). Metabolic endotoxemia initiates obesity and insulin resistance. Diabetes.

[24] Cai D, Yuan M, Frantz DF, Melendez PA, Hansen L, Lee J (2005). Local and systemic insulin resistance resulting from hepatic activation of IKK-beta and NF-kappaB. Nat Med.

[25] Sell H, Habich C, Eckel J (2012). Adaptive immunity in obesity and insulin resistance. Nat Rev Endocrinol.

[26] Lumeng CN, Saltiel AR (2011). Inflammatory links between obesity and metabolic disease. J Clin Invest.

[27] Tanti JF, Ceppo F, Jager J, Berthou F (2012). Implication of inflammatory signaling pathways in obesity-induced insulin resistance. Front Endocrinol (Lausanne).

[28] Piya MK, McTernan PG, Kumar S (2013). Adipokine inflammation and insulin resistance: the role of glucose, lipids and endotoxin. J Endocrinol.

[29] Tack CJ, Stienstra R, Joosten LA, Netea MG (2012). Inflammation links excess fat to insulin resistance: the role of the interleukin-1 family. Immunol Rev.

[30] Larsen CM, Faulenbach M, Vaag A, Volund A, Ehses JA, Seifert B (2007). Interleukin-1-receptor antagonist in type 2 diabetes mellitus. N Engl J Med.

[31] Grant RW, Dixit VD (2013). Mechanisms of disease: inflammasome activation and the development of type 2 diabetes. Front Immunol.

[32] Clarke G, Stilling RM, Kennedy PJ, Stanton C, Cryan JF, Dinan TG (2014). Minireview: Gut microbiota: the neglected endocrine organ. Mol Endocrinol.

[33] Turnbaugh PJ, Ley RE, Mahowald MA, Magrini V, Mardis ER, Gordon JI (2006). An obesity-associated gut microbiome with increased capacity for energy harvest. Nature.

[34] Samuel BS, Shaito A, Motoike T, Rey FE, Backhed F, Manchester JK (2008). Effects of the gut microbiota on host adiposity are modulated by the short-chain fatty-acid binding G protein-coupled receptor, Gpr41. Proc Natl Acad Sci U S A.

[35] Larsson E, Tremaroli V, Lee YS, Koren O, Nookaew I, Fricker A (2012). Analysis of gut microbial regulation of host gene expression along the length of the gut and regulation of gut microbial ecology through MyD88. Gut.

[36] Musso G, Gambino R, Cassader M (2011). Interactions between gut microbiota and host metabolism predisposing to obesity and diabetes. Annu Rev Med.

[37] Gibson GR, Probert HM, Loo JV, Rastall RA, Roberfroid MB (2004). Dietary modulation of the human colonic microbiota: updating the concept of prebiotics. Nutr Res Rev.

[38] Winder WW, Hardie DG (1999). AMP-activated protein kinase, a metabolic master switch: possible roles in type 2 diabetes. Am J Physiol.

[39] Bellahcene M, O'Dowd JF, Wargent ET, Zaibi MS, Hislop DC, Ngala RA (2013). Male mice that lack the G-protein-coupled receptor GPR41 have low energy expenditure and increased body fat content. Br J Nutr.

[40] Smith PM, Howitt MR, Panikov N, Michaud M, Gallini CA, Bohlooly YM (2013). The microbial metabolites, short-chain fatty acids, regulate colonic Treg cell homeostasis. Science.

[41] Parseus A, Sommer N, Sommer F, Caesar R, Molinaro A, Stahlman M, et al. Microbiota-induced obesity requires farnesoid X receptor. Gut. 2016. doi:10.1136/gutjnl-2015-310283. 10.1136/gutjnl-2015-310283 PMC5534765 26740296

[42] Noga AA, Vance DE (2003). A gender-specific role for phosphatidylethanolamine N-methyltransferase-derived phosphatidylcholine in the regulation of plasma high density and very low density lipoproteins in mice. J Biol Chem.

[43] Cole LK, Vance JE, Vance DE (1821). Phosphatidylcholine biosynthesis and lipoprotein metabolism. Biochim Biophys Acta.

[44] Dumas ME, Barton RH, Toye A, Cloarec O, Blancher C, Rothwell A (2006). Metabolic profiling reveals a contribution of gut microbiota to fatty liver phenotype in insulin-resistant mice. Proc Natl Acad Sci U S A.

[45] Ley RE, Backhed F, Turnbaugh P, Lozupone CA, Knight RD, Gordon JI (2005). Obesity alters gut microbial ecology. Proc Natl Acad Sci U S A.

[46] Murphy EF, Cotter PD, Healy S, Marques TM, O'Sullivan O, Fouhy F (2010). Composition and energy harvesting capacity of the gut microbiota: relationship to diet, obesity and time in mouse models. Gut.

[47] Ridaura VK, Faith JJ, Rey FE, Cheng J, Duncan AE, Kau AL (2013). Gut microbiota from twins discordant for obesity modulate metabolism in mice. Science.

[48] De Vadder F, Kovatcheva-Datchary P, Goncalves D, Vinera J, Zitoun C, Duchampt A (2014). Microbiota-generated metabolites promote metabolic benefits via gut-brain neural circuits. Cell.

[49] Duncan SH, Lobley GE, Holtrop G, Ince J, Johnstone AM, Louis P (2008). Human colonic microbiota associated with diet, obesity and weight loss. Int J Obes (Lond).

[50] Shi H, Kokoeva MV, Inouye K, Tzameli I, Yin H, Flier JS (2006). TLR4 links innate immunity and fatty acid-induced insulin resistance. J Clin Invest.

[51] Le Chatelier E, Nielsen T, Qin J, Prifti E, Hildebrand F, Falony G (2013). Richness of human gut microbiome correlates with metabolic markers. Nature.

[52] David LA, Maurice CF, Carmody RN, Gootenberg DB, Button JE, Wolfe BE (2014). Diet rapidly and reproducibly alters the human gut microbiome. Nature.

[53] Chassaing B, Gewirtz AT (2016). Has provoking microbiota aggression driven the obesity epidemic?. Bioessays.

[54] Cotillard A, Kennedy SP, Kong LC, Prifti E, Pons N, Le Chatelier E (2013). Dietary intervention impact on gut microbial gene richness. Nature.

[55] Vreugdenhil AC, Rousseau CH, Hartung T, Greve JW, van't Veer C, Buurman WA (2003). Lipopolysaccharide (LPS)-binding protein mediates LPS detoxification by chylomicrons. J Immunol.

[56] Neal MD, Leaphart C, Levy R, Prince J, Billiar TR, Watkins S (2006). Enterocyte TLR4 mediates phagocytosis and translocation of bacteria across the intestinal barrier. J Immunol.

[57] Vijay-Kumar M, Aitken JD, Carvalho FA, Cullender TC, Mwangi S, Srinivasan S (2010). Metabolic syndrome and altered gut microbiota in mice lacking Toll-like receptor 5. Science.

[58] Ghoshal S, Witta J, Zhong J, de Villiers W, Eckhardt E (2009). Chylomicrons promote intestinal absorption of lipopolysaccharides. J Lipid Res.

[59] Verges B, Duvillard L, Lagrost L, Vachoux C, Garret C, Bouyer K (2014). Changes in lipoprotein kinetics associated with type 2 diabetes affect the distribution of lipopolysaccharides among lipoproteins. J Clin Endocrinol Metab.

[60] Cani PD, Bibiloni R, Knauf C, Waget A, Neyrinck AM, Delzenne NM (2008). Changes in gut microbiota control metabolic endotoxemia-induced inflammation in high-fat diet-induced obesity and diabetes in mice. Diabetes.

[61] Harte AL, Varma MC, Tripathi G, McGee KC, Al-Daghri NM, Al-Attas OS (2012). High fat intake leads to acute postprandial exposure to circulating endotoxin in type 2 diabetic subjects. Diabetes Care.

[62] Ghanim H, Abuaysheh S, Sia CL, Korzeniewski K, Chaudhuri A, Fernandez-Real JM (2009). Increase in plasma endotoxin concentrations and the expression of Toll-like receptors and suppressor of cytokine signaling-3 in mononuclear cells after a high-fat, high-carbohydrate meal: implications for insulin resistance. Diabetes Care.

[63] Russell WR, Hoyles L, Flint HJ, Dumas ME (2013). Colonic bacterial metabolites and human health. Curr Opin Microbiol.

[64] DeMoss RD, Moser K (1969). Tryptophanase in diverse bacterial species. J Bacteriol.

[65] Russell WR, Duncan SH, Scobbie L, Duncan G, Cantlay L, Calder AG (2013). Major phenylpropanoid-derived metabolites in the human gut can arise from microbial fermentation of protein. Mol Nutr Food Res.

[66] Venkatesh M, Mukherjee S, Wang H, Li H, Sun K, Benechet AP (2014). Symbiotic bacterial metabolites regulate gastrointestinal barrier function via the xenobiotic sensor PXR and Toll-like receptor 4. Immunity.

[67] Ramadoss P, Marcus C, Perdew GH (2005). Role of the aryl hydrocarbon receptor in drug metabolism. Expert Opin Drug Metab Toxicol.

[68] Cani PD, Osto M, Geurts L, Everard A (2012). Involvement of gut microbiota in the development of low-grade inflammation and type 2 diabetes associated with obesity. Gut Microbes.

[69] Li YT, Cai HF, Wang ZH, Xu J, Fang JY (2016). Systematic review with meta-analysis: long-term outcomes of faecal microbiota transplantation for Clostridium difficile infection. Aliment Pharmacol Ther.

[70] Colman RJ, Rubin DT (2014). Fecal microbiota transplantation as therapy for inflammatory bowel disease: a systematic review and meta-analysis. J Crohns Colitis.

[71] Vrieze A, Van Nood E, Holleman F, Salojarvi J, Kootte RS, Bartelsman JF (2012). Transfer of intestinal microbiota from lean donors increases insulin sensitivity in individuals with metabolic syndrome. Gastroenterology.

[72] Kobyliak N, Conte C, Cammarota G, Haley AP, Styriak I, Gaspar L (2016). Probiotics in prevention and treatment of obesity: a critical view. Nutr Metab (Lond).

[73] Hill C, Guarner F, Reid G, Gibson GR, Merenstein DJ, Pot B (2014). Expert consensus document. The International Scientific Association for Probiotics and Prebiotics consensus statement on the scope and appropriate use of the term probiotic. Nat Rev Gastroenterol Hepatol.

[74] Cani PD, Knauf C, Iglesias MA, Drucker DJ, Delzenne NM, Burcelin R (2006). Improvement of glucose tolerance and hepatic insulin sensitivity by oligofructose requires a functional glucagon-like peptide 1 receptor. Diabetes.

[75] Delzenne NM, Kok N (2001). Effects of fructans-type prebiotics on lipid metabolism. Am J Clin Nutr.

[76] van Hoffen E, Ruiter B, Faber J, M'Rabet L, Knol EF, Stahl B (2009). A specific mixture of short-chain galacto-oligosaccharides and long-chain fructo-oligosaccharides induces a beneficial immunoglobulin profile in infants at high risk for allergy. Allergy.

[77] Daddaoua A, Puerta V, Requena P, Martinez-Ferez A, Guadix E, de Medina FS (2006). Goat milk oligosaccharides are anti-inflammatory in rats with hapten-induced colitis. J Nutr.

[78] Cani PD, Neyrinck AM, Fava F, Knauf C, Burcelin RG, Tuohy KM (2007). Selective increases of bifidobacteria in gut microflora improve high-fat-diet-induced diabetes in mice through a mechanism associated with endotoxaemia. Diabetologia.

[79] Delzenne NM, Kok N (1998). Effect of non-digestible fermentable carbohydrates on hepatic fatty acid metabolism. Biochem Soc Trans.

[80] Tazoe H, Otomo Y, Karaki S, Kato I, Fukami Y, Terasaki M (2009). Expression of short-chain fatty acid receptor GPR41 in the human colon. Biomed Res.

[81] Karaki S, Tazoe H, Hayashi H, Kashiwabara H, Tooyama K, Suzuki Y (2008). Expression of the short-chain fatty acid receptor, GPR43, in the human colon. J Mol Histol.

[82] Zhou J, Hegsted M, McCutcheon KL, Keenan MJ, Xi X, Raggio AM (2006). Peptide YY and proglucagon mRNA expression patterns and regulation in the gut. Obesity (Silver Spring).

[83] Zhou J, Martin RJ, Tulley RT, Raggio AM, McCutcheon KL, Shen L (2008). Dietary resistant starch upregulates total GLP-1 and PYY in a sustained day-long manner through fermentation in rodents. Am J Physiol Endocrinol Metab.

[84] Delzenne NM, Cani PD, Neyrinck AM (2007). Modulation of glucagon-like peptide 1 and energy metabolism by inulin and oligofructose: experimental data. J Nutr.

[85] Garidou L, Pomie C, Klopp P, Waget A, Charpentier J, Aloulou M (2015). The gut microbiota regulates intestinal CD4 T cells expressing RORgammat and controls metabolic disease. Cell Metab.

[86] Ivey KL, Hodgson JM, Kerr DA, Lewis JR, Thompson PL, Prince RL (2014). The effects of probiotic bacteria on glycaemic control in overweight men and women: a randomised controlled trial. Eur J Clin Nutr.

[87] Rajkumar H, Mahmood N, Kumar M, Varikuti SR, Challa HR, Myakala SP (2014). Effect of probiotic (VSL#3) and omega-3 on lipid profile, insulin sensitivity, inflammatory markers, and gut colonization in overweight adults: a randomized, controlled trial. Mediators Inflamm.

[88] Sanchez M, Darimont C, Drapeau V, Emady-Azar S, Lepage M, Rezzonico E (2014). Effect of Lactobacillus rhamnosus CGMCC1.3724 supplementation on weight loss and maintenance in obese men and women. Br J Nutr.

[89] Parnell JA, Reimer RA (2009). Weight loss during oligofructose supplementation is associated with decreased ghrelin and increased peptide YY in overweight and obese adults. Am J Clin Nutr.

[90] Cani PD, Lecourt E, Dewulf EM, Sohet FM, Pachikian BD, Naslain D, De Backer F, Neyrinck AM, Delzenne NM (2009). Gut microbiota fermentation of prebiotics increases satietogenic and incretin gut peptide production with consequences for appetite sensation and glucose response after a meal. Am J Clin Nutr.

[91] Vulevic J, Juric A, Tzortzis G, Gibson GR (2013). A mixture of trans-galactooligosaccharides reduces markers of metabolic syndrome and modulates the fecal microbiota and immune function of overweight adults. J Nutr.

[92] Vulevic J, Drakoularakou A, Yaqoob P, Tzortzis G, Gibson GR (2008). Modulation of the fecal microflora profile and immune function by a novel trans-galactooligosaccharide mixture (B-GOS) in healthy elderly volunteers. Am J Clin Nutr.

[93] Zhang C, Yin A, Li H, Wang R, Wu G, Shen J (2015). Dietary modulation of gut microbiota contributes to alleviation of both genetic and simple obesity in children. EBioMedicine.

[94] Ussar S, Griffin NW, Bezy O, Fujisaka S, Vienberg S, Softic S (2015). Interactions between gut microbiota, host genetics and diet modulate the predisposition to obesity and metabolic syndrome. Cell Metab.

